# Enhancing diagnosis of benign lesions and lung cancer through ensemble text and breath analysis: a retrospective cohort study

**DOI:** 10.1038/s41598-024-59474-w

**Published:** 2024-04-16

**Authors:** Hao Wang, Yinghua Wu, Meixiu Sun, Xiaonan Cui

**Affiliations:** 1 Institute of Biomedical Engineering, Chinese Academy of Medical Sciences & Peking Union Medical College, Tianjin, China; 2 Department of Radiology, Key Laboratory of Cancer Prevention and Therapy, National Clinical Research Centre of Cancer, Tianjin Medical University Cancer Institute and Hospital, Tianjin, 300060 China; 3 Engineering Research Center of Pulmonary and Critical Care Medicine Technology and Device Ministry of Education, Chinese Academy of Medical Sciences & Peking Union Medical College, Tianjin, China

**Keywords:** Breath analysis, Natural language processing, Lung cancer, Benign lesion, ensemble model, Lung cancer, Mass spectrometry

## Abstract

Early diagnosis of lung cancer (LC) can significantly reduce its mortality rate. Considering the limitations of the high false positive rate and reliance on radiologists’ experience in computed tomography (CT)–based diagnosis, a multi-modal early LC screening model that combines radiology with other non-invasive, rapid detection methods is warranted. A high-resolution, multi-modal, and low-differentiation LC screening strategy named ensemble text and breath analysis (ETBA) is proposed that ensembles radiology report text analysis and breath analysis. In total, 231 samples (140 LC patients and 91 benign lesions [BL] patients) were screened using proton transfer reaction–time of flight–mass spectrometry and CT screening. Participants were randomly assigned to a training set and a validation set (4:1) with stratification. The report section of the radiology reports was used to train a text analysis (TA) model with a natural language processing algorithm. Twenty-two volatile organic compounds (VOCs) in the exhaled breath and the prediction results of the TA model were used as predictors to develop the ETBA model using an extreme gradient boosting algorithm. A breath analysis model was developed based on the 22 VOCs. The BA and TA models were compared with the ETBA model. The ETBA model achieved a sensitivity of 94.3%, a specificity of 77.3%, and an accuracy of 87.7% with the validation set. The radiologist diagnosis performance with the validation set had a sensitivity of 74.3%, a specificity of 59.1%, and an accuracy of 68.1%. High sensitivity and specificity were obtained by the ETBA model compared with radiologist diagnosis. The ETBA model has the potential to provide sensitivity and specificity in CT screening of LC. This approach is rapid, non-invasive, multi-dimensional, and accurate for LC and BL diagnosis.

## Introduction

Lung cancer (LC) has a relatively high incidence and mortality among cancers^1^. The major cause of its high mortality is that most cases are advanced stage at initial diagnosis^2^. According to clinical statistics, the 5-years survival rate of stage IV non-small cell lung carcinoma (NSCLC) is only 5%^2^ compared with 60% for stage I NSCLC subjects who have received surgery^3^. Therefore, effective early screening strategies can play a crucial role in reducing LC mortality. Currently, the early screening strategy adopted in clinical practice is computed tomography (CT), which has been proven to contribute 20% to the relative risk reduction of LC death^4^. However, this strategy relies on radiologists’ experience, which has no common standard^5^. An article reported that professional radiologists only achieved 46.53% diagnostic sensitivity due to false and missed detections caused by reading a large number of scans^6^. In addition, as LC and benign lesions (BL) are asymptomatic, diagnosis by CT results in a high false positive rate (FPR)^7^. In order to enhance the diagnostic accuracy of lung cancer and benign tumors, thereby accurately distinguishing early-stage lung cancer patients from those with benign tumors, and ultimately improving the 5-years survival rate of lung cancer patients, we have developed an ensemble model strategy utilizing artificial intelligence which exhaled breath analysis with imaging report analysis.

With the refinement of electronic health systems (EHSs) and the rapid development of natural language processing (NLP), text analysis (TA) of radiology reports has been increasingly studied for the early screening of LC^8^. Radiology reports that are generated by expert radiologists and stored in EHSs consist of the report sections that record the imaging findings and the impression section that records the final impression^9^. TA with NLP models has demonstrated the feasibility of recapitulating the outcome annotations of radiology reports of non-small cell LC, thus serving as an extension of low-dose CT screening^10^. Nobel et al. used radiology reports to classify lung tumors automatically according to the 8th TNM classification system^11^. Although TA on radiology reports that contain a comprehensive description of the CT findings can achieve a diagnostic sensitivity of 77.3% compared with the 51.5% achieved by the radiologist diagnostic model^12^, this approach has the limitations of a high FPR^13^.

Breath analysis (BA) is a novel, promising diagnosis method based on metabolic processes at the tissue level^14^. Exhaled breath from humans contains thousands of low-concentration volatile organic compounds (VOCs), which can be detected by special techniques (e.g., gas chromatography–mass spectrometry (GC–MS) and proton transfer reaction–mass spectrometry (PTR–MS))^16^. BA has shown effectiveness in diagnosing LC according to many reports^17^. Most studies using the BA method have been able to distinguish LC and healthy controls^18^. It is worth noting that the majority of cases have pulmonary nodules (PNs) in the early screening population. In 2019, Phillips et al. used biomarkers of Mass Abnormalities in Gaseous Ions with Imaging Correlates and attained 75.4% sensitivity and 85.0% specificity in distinguishing LC from PNs^21^. In 2021, Chen et al. identified LC and BL using BA and thermal desorption–GC–MS, with the area under the receiver operating characteristic (ROC) curve (AUC) of 0.809^22^. As BA can effectively identify LC from PNs and help make overall judgments at the tissue level, we propose a high-resolution and comprehensive strategy named ensemble text and breath analysis (ETBA) that combines TA and BA analysis to diagnose BL and LC.

## Methods

### Participants

**Figure 1:**
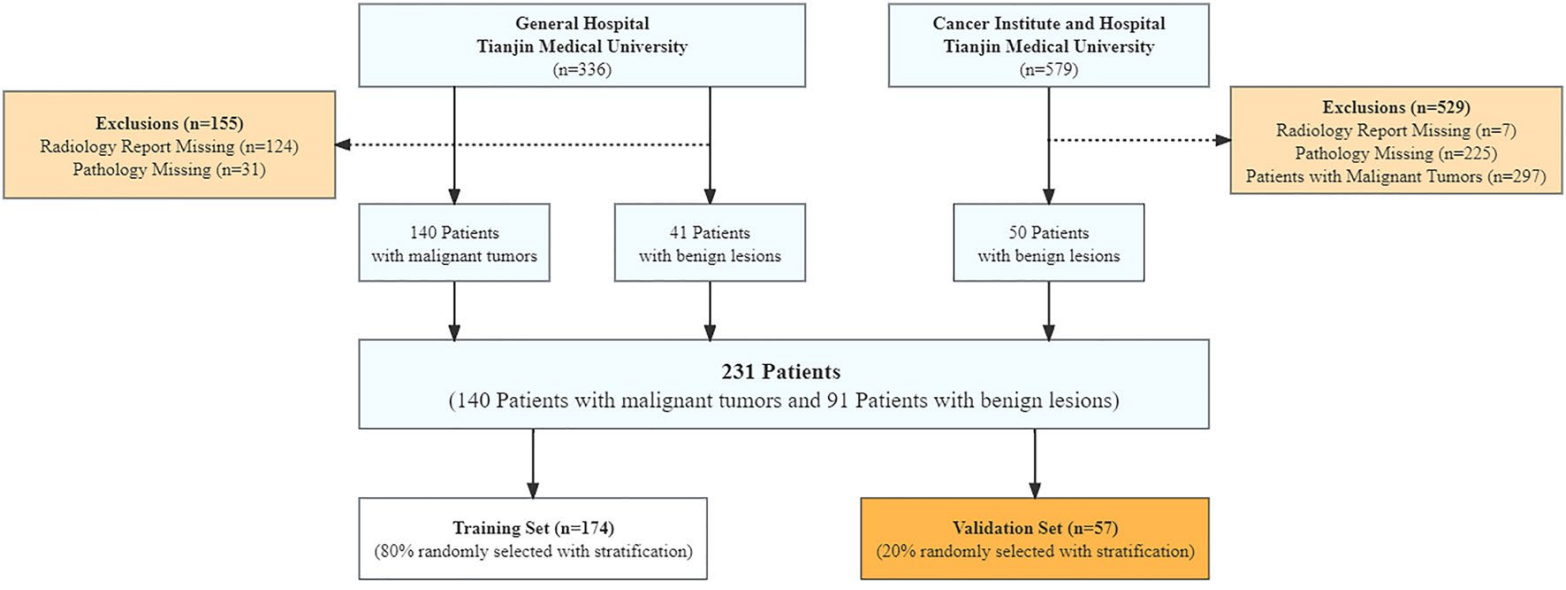
Participant recruitment, exhaled breath analysis, and data analysis process.

In this study, 231 adults consisting of 140 LC patients and 91 BL controls were recruited with the method of randomized controlled trial. Among them, 181 adults (140 LC patients and 41 BL patients) were recruited from the pulmonary oncology department of Tianjin Medical University’s General Hospital between October 2020 and June 2021. To balance the sample size, 50 BL adults were recruited from the pulmonary oncology department of Tianjin Medical University’s Cancer Institute and Hospital between February 2019 and September 2019. The recruitment process is shown in Fig. 1. All participants were examined by CT, and LC status was confirmed by pathology. The demographic, pathological, and stage characteristics are shown in Table 1. The smoking status was delineated as never smoked, ex-smoker, and currently smoking. Smoking denoted at least one cigarette every day, which continued on average for more than 6 months; and an ex-smoker was defined as having quit smoking 4 or more months prior to sampling. The radiology reports were written as standard. Participants were randomly split into the training set and validation set (4:1) with stratification. The exclusion criteria were as follows: (i) participants under 18 years of age; (ii) participants with tumor history; (iii) participants who had received chemotherapy (with anti-cancer drugs), immunotherapy, hormone therapy, or radiation therapy; and (iv) women who were pregnant.

**Table 1:** Characteristics of the participants.

Characteristics	Training set		Validation set		*P*
	Lung cancer (n = 105)	Benign lesions (n = 69)	Lung cancer (n = 35)	Benign lesions (n = 22)	
Age	61.93 ± 9.17	57.96 ± 10.48	60.86 ± 9.42	58.95 ± 8.00	0.69
BMI	23.77 ± 4.05	24.17 ± 3.02	23.27 ± 2.88	24.84 ± 3.26	0.91
Male (%)	42 (40.0%)	38 (55.1%)	11 (31.4%)	10 (45.5%)	0.21
Fasting (%)	11 (10.5%)	17 (24.6%)	3 (8.6%)	7 (31.8%)	0.95
Smoking (%)					0.60
Smokers	6 (5.7%)	6 (8.7%)	2 (5.7%)	0 (0%)	
Ex-smokers	35 (33.3%)	23 (33.3%)	9 (25.7%)	8 (36.4%)	
Non-smokers	64 (61.0%)	40 (58.0%)	24 (68.6%)	14 (63.6%)	
Category (%)					0.68
Adenocarcinoma	85 (81.0%)	NA	33 (94.3%)	NA	
Squamous cell carcinoma	10 (9.5%)	NA	1 (2.9%)	NA	
Small-cell lung cancer	3 (2.9%)	NA	1 (2.9%)	NA	
Missing	7 (6.7%)	NA	0 (0%)	NA	
Stage (%)					0.37
I	73 (69.5%)	NA	28 (80.0%)	NA	
II	2 (1.9%)	NA	1 (2.9%)	NA	
III	20 (19.0%)	NA	4 (11.4%)	NA	
IV	6 (5.7%)	NA	1 (2.9%)	NA	
Missing	4 (3.8%)	NA	1 (2.9%)	NA	

### Participants’ characteristics sampling procedures

Each participant was sampled in an individualized room equipped with a dedicated sampling apparatus. Prior to the sampling procedure, participants provided self-reported data pertaining to their fasting status, tobacco consumption habits, medication intake, and any previous history of pulmonary disorders. A history of pulmonary disease was defined by an affirmative response to the query, “Have you ever been diagnosed with a lung disease?” Medication utilization was characterized as the intake of any pharmaceutical agent, encompassing sprays, tablets, capsules, and herbal concoctions, within the preceding fortnight. Fasting status was determined by a positive response to the question, “Have you consumed breakfast today?” Smoking status was categorized into three distinct groups: never smokers, former smokers, and current smokers. Smoking was defined as the consumption of a minimum of one cigarette daily persisting for a duration exceeding or averaging 6 months. A former smoker was identified as an individual who had abstained from smoking for a minimum duration of 4 months prior to the sampling event. The amount of smoking was determined by counting the number of cigarettes smoked per day.

### Breath sampling procedures

We employed the buffered end-tidal (BET) sampling system (Ionicon Analytik GmbH, Innsbruck, Austria) integrated on the PTR–TOF–MS 1000 (Ionicon Analytik GmbH) as the designated breath sampling apparatus for this experiment. The BET sampling system offers two unique benefits: it captures the end-tidal fraction of the exhaled breath gas accurately and ensures that the test subjects sustain a regular breathing rhythm following exhalation. This system enables the assessment of endogenous compounds produced from the alveolar blood–gas exchange and diminishes the likelihood of hyperventilation. Prior to exhalation, each participant was instructed to take a deep breath and then exhale into the BET sampling system with a disposable and sterile mouthpiece (Polypropylene; Art. Nr. 31-30-0022, Germany). During the exhalation process, no breath changes were permitted, ensuring that each measurement yielded a consistent and smooth waveform. Each participant underwent three exhalation trials to minimize error. Before initiating each trial, it was ensured that the waveform displayed on the instrument returned to its baseline value, thereby eliminating any influence from the preceding exhalation trial.

### Breath sample detection

We selected the PTR–TOF–MS 1000 coupled with BET online sampling as our instrument for breath sample detection. This state-of-the-art system provides real-time quantitative analysis of the end-tidal fraction of the exhaled breath gas, with an ultra-low detection limit (LoD < 10 pptv) and high resolution (> 2000 m/△m). To counteract the condensation of humidified breath gas, the buffer tube was maintained at a constant 80 °C using a heating mechanism. Subsequently, the gas was channeled into the ionization section via the instrument’s inlet line. Within this section, ionized molecules underwent separation based on their mass-to-charge ratio (m/z) before being detected. The drift tube operated at a pressure and temperature of 2.3 mbar and 70 °C, respectively, and it was subjected to an electric drift field of 600 V. A total of 318 features (m/z) were extracted from the acquired spectrum of each exhaled breath sample. The stabilized band was isolated, and its average concentration was computed. For this study, a model was established using 22 specific VOCs (listed in Extended Data Table 1).

### TA model construction

**Figure 2:**
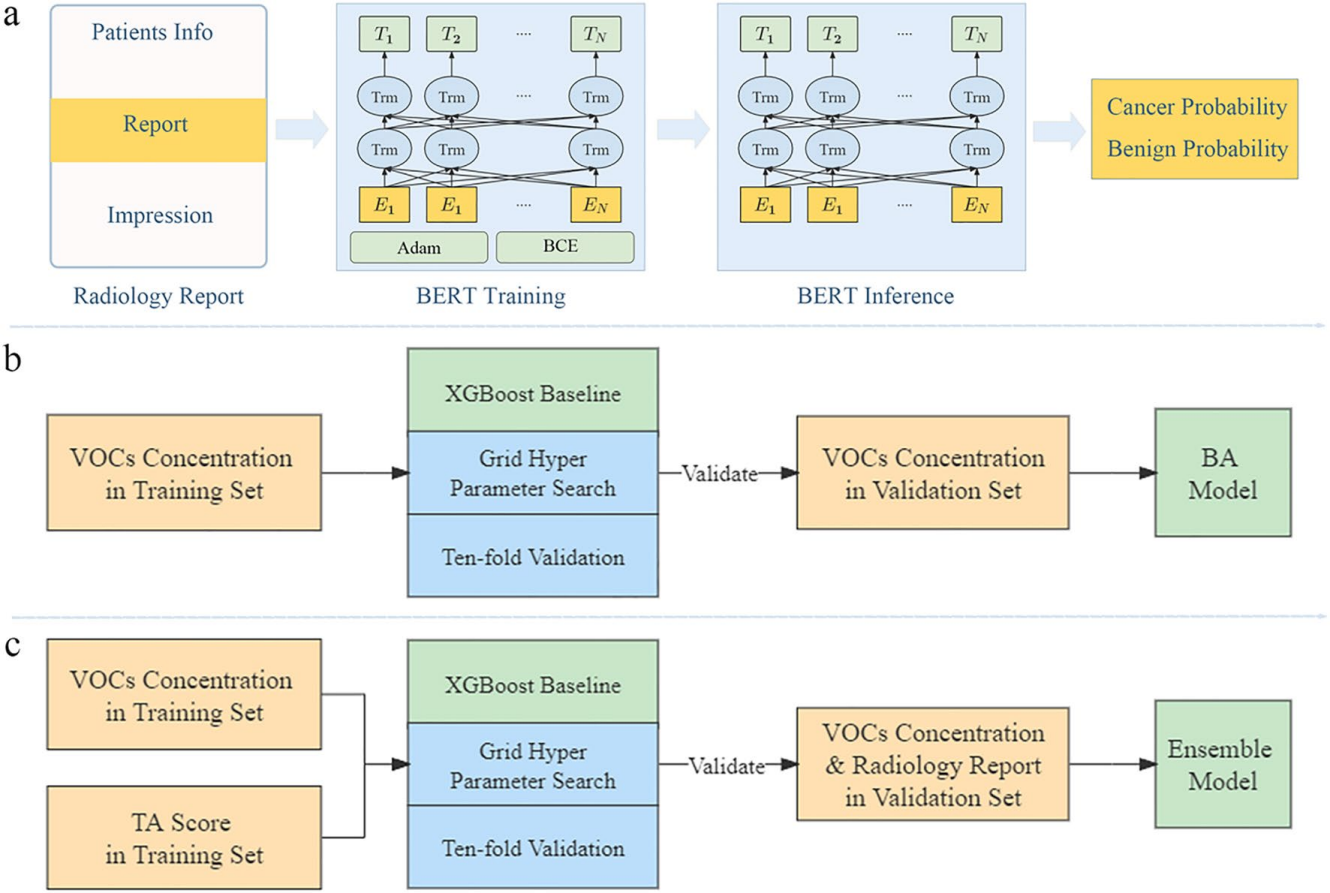
Model structure. (**a**) TA model structure. (**b**) BA model structure. (**c**) ETBA model structure.

The intended core function of the TA model is to play the role of a radiologist in identifying LC and PNs based on a radiology report, which could be treated as a text classification problem. With a multi-headed attention mechanism, transformer-based NLP models are widely implemented in the medical field. In this study, bidirectional encoder representations from transformers (BERT) were selected to construct the TA model. The structure of the TA model is shown in Fig. 2a. BERT extracts text features and decreases the loss function during training. Given a radiology report as the input text, its prediction score encoded in one-hot format can be accessed by the BERT model after training and saving.

The loss function and learning rate optimization are critical parameters for training. In this study, the binary cross entropy loss function was selected to avoid gradient disappearance. The Adam optimizer automatically adapts the learning rate to find the minimum of the loss function. Before training, the impression section of the radiology report that was recorded in the EHS was removed to avoid any influence on the diagnostic tendency of the radiologist. We set the F1 score as the training criterion and trained and validated the BERT model for 200 epochs. The best weighting parameters were saved.

### BA model and ETBA model construction

For classified tasks with simple and quantifiable features, machine learning is an appropriate approach. In this study, we selected the extreme gradient boosting (XGBoost) classification model with a gbtree kernel to distinguish LC and BL. The core of XGBoost is node splitting and depth growth to construct a decision tree that has the best performance on the classified task. Given the features (VOCs as the features for the BA model; VOCs and the TA model score as the features for the ETBA model) as the input, the prediction score was obtained using the XGBoost model.

The max depth, gamma value, and lambda value are critical for model training. Grid parameter search was adopted to find the best combination of the parameters that were already in the set search scope. Before training, min–max and square root normalization were applied to the original data as a preprocessing step. Ten-fold cross-validation was performed on the training set to find the best parameter combination according to the AUC. After testing, the best values of the max depth, gamma, and lambda were found to be 5, 0.15, and 5, respectively. Therefore, we reset the parameters, trained the model on the whole training set, and validated it. The construction flow of the BA and ETBA models is shown in Fig. 2b, c.

### Statistical analysis

Accuracy, sensitivity, and specificity were used to evaluate the diagnostic model. The AUC and ROC were also calculated to evaluate the classification performance of the diagnostic model. Feature importance was adopted to evaluate the classification ability for each VOCs and TA score. The heat map of the confusion matrix reflected the effectiveness of the ETBA model.

### Safety precautions

The PTR–TOF–MS was placed in a separate room, and participants were guided through breath collection by a professional sampler. There was only one participant in the room at a time. The sampler was required to wear a mask for safety. The participant blew into the BET sampling system through a disposable mouthpiece, and there was no direct contact between the BET sampling system and the subject. Any gas remaining after collection by the BET sampling system was removed by an extractor.

### Ethics approval

The study was conducted in compliance with the principles outlined in the Declaration of Helsinki and received approval from the Ethics Committees of the Cancer Institute and Hospital, Tianjin Medical University, as well as Tianjin Medical University General Hospital. This trial was registered with the Institutional Review Board of the Chinese Clinical Trial Registry (registration number: chiCTR1900023659), and all procedures adhered to pertinent guidelines and regulations. Informed consent was obtained from all participants.

## Results

### Characteristics of the participants

A total of 231 participants were recruited for this analysis, comprising 140 LC and 91 BL patients. In the total set, 101 were men, and the mean and standard deviation of the age were 60.3 and 9.8 years, respectively. Participants were split into the training set (105 LC and 69 BL participants) and the validation set (35 LC and 22 BL participants) with stratification.

### PTR–TOF–MS detection

**Figure 3:**
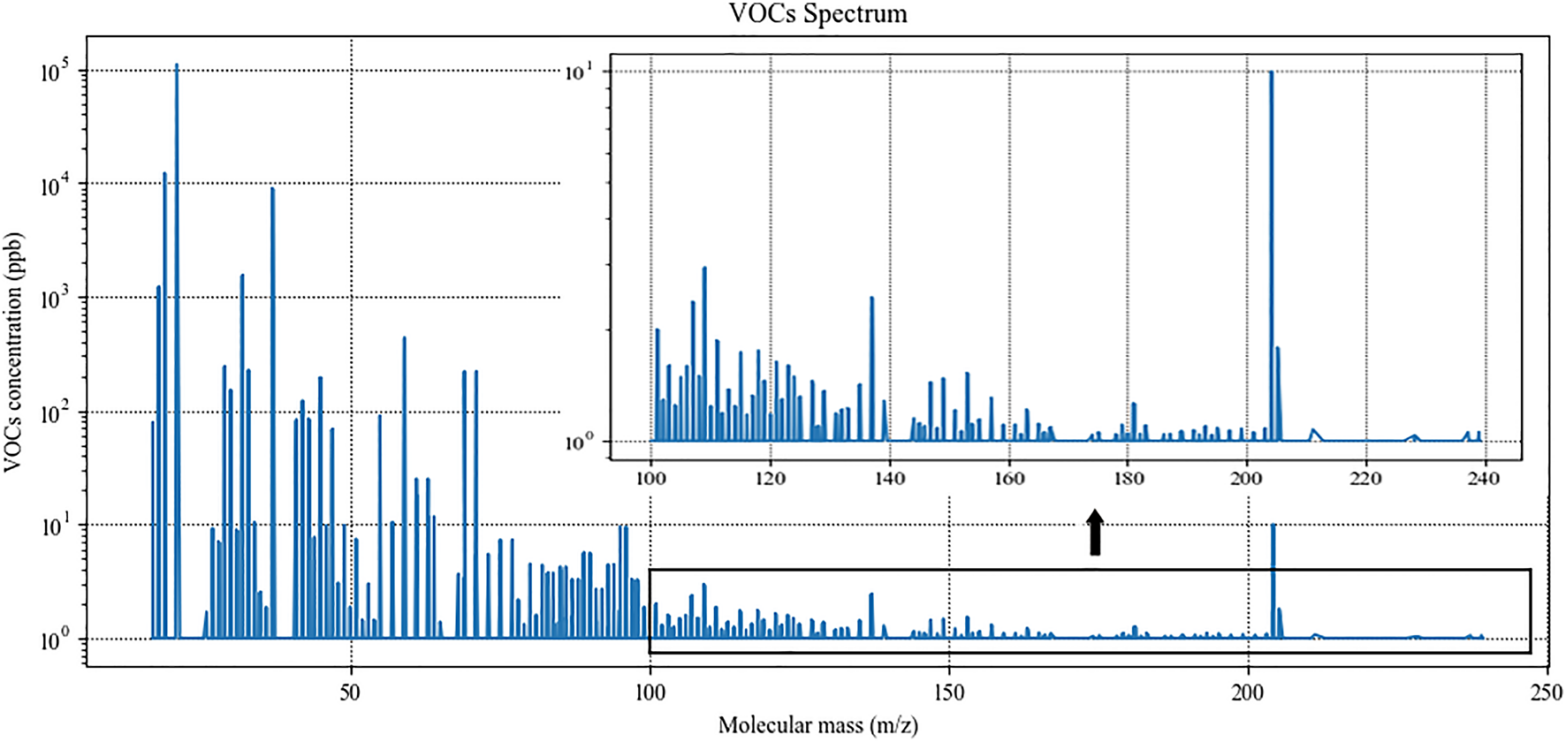
VOCs detection spectrum of the participants.

For each participant, the detection was performed three times, and the calculated stable waveform average value was calculated and considered as the concentration of the VOCs. The spectrum of detection is shown in Fig. 3. A total of 318 VOCs were inspected, and we extracted 22 endogenous VOCs that had stable and low-noise waveforms for the model’s construction.

### Evaluation of the models with the validation set

**Figure 4:**
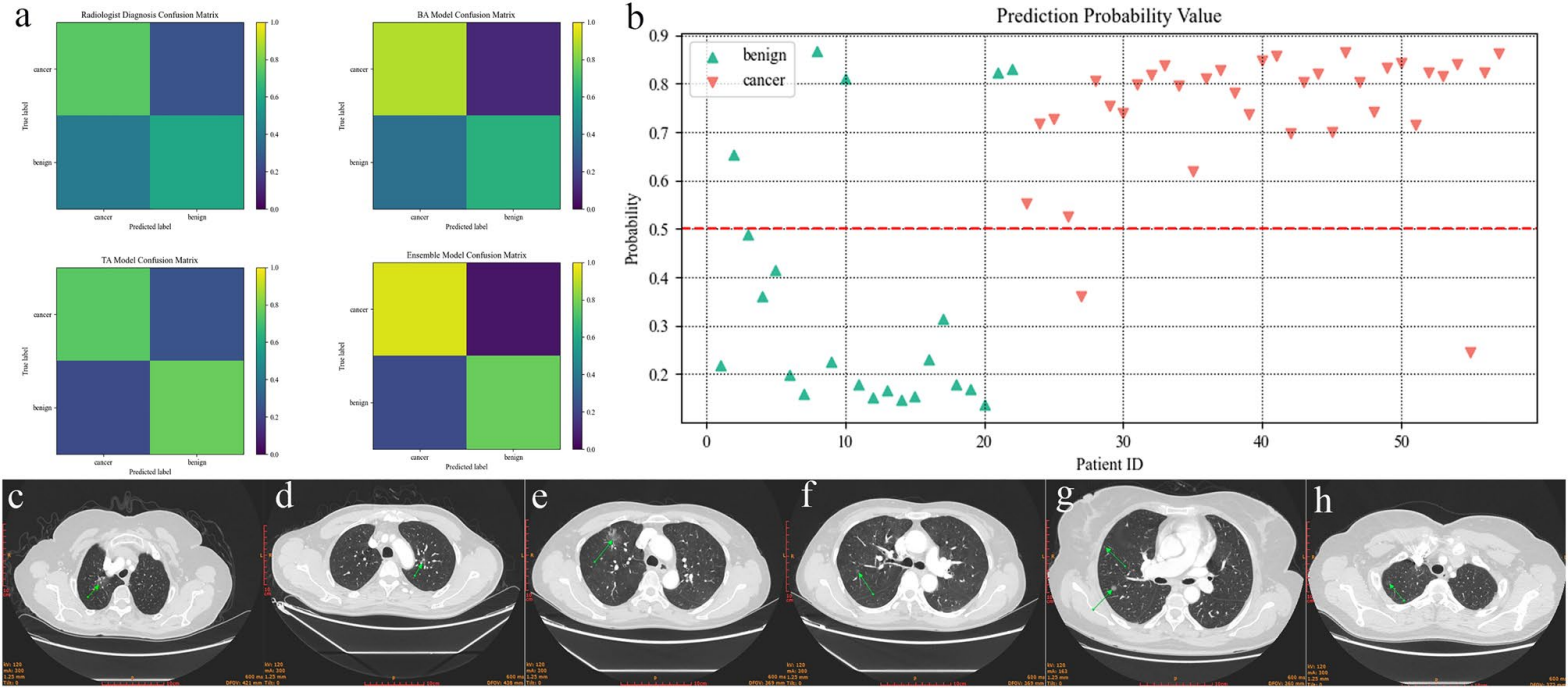
Evaluation of the ETBA model. (**a**) Confusion matrix of the radiologist diagnosis, BA model, TA model, and ETBA model. (**b**) Prediction score by the ETBA model for each participant. (**c**–**e**) Radiographs of the cases for which the radiologist suspected inflammation or the TA model made an error or reported uncertain diagnosis and for which the ETBA model predicted correctly. (**f**–**h**) Radiographs of the cases for which the radiologist diagnosed small ground glass nodules and conservative treatment or the TA model made an error or reported uncertain diagnosis and for which the ETBA model predicted correctly (green arrow shows tumor position).

The radiologist diagnosis attained a sensitivity of 74.3%, a specificity of 59.1%, and an accuracy of 68.1%. The TA model attained a sensitivity of 74.3%, a specificity of 77.3%, an accuracy of 75.4%, and an AUC of 0.838 (95% confidence interval [CI] from 0.712 to 0.879). The BA model achieved a sensitivity of 88.6%, a specificity of 63.6%, an accuracy of 79.0%, and an AUC of 0.779 (95% CI from 0.742 to 0.842). The ETBA model obtained a sensitivity of 94.3%, a specificity of 77.3%, an accuracy of 87.7%, and an AUC of 0.845 (95% CI from 0.738 to 0.919). These indicators demonstrated the performance of the models. The AUC reflected the robustness of the models. The evaluation of the models is shown in Table 2. The evaluation of the models with the training set is shown in Extended Data Table 2. The confusion matrix of the models is shown in Fig. 4a. The prediction scores of the models for each participant in the validation set are shown in Fig. 4b. Some LC cases that the radiologist and the TA model erroneously labeled and cases of uncertain diagnosis that the ETBA model diagnosed correctly and certainly are shown in Fig. 4c–h. The major reasons leading to misdiagnosis or uncertain diagnosis were similarity to inflammation or small nodule size, which is considered to warrant conservative treatment.

**Table 2:** Sensitivity, specificity, and accuracy of the models and of the manual diagnosis with the validation set.

Method	Sensitivity (%)	Specificity (%)	Accuracy (%)
Radiologist diagnosis	74.3	59.1	68.1
TA	74.3	77.3	75.4
BA	88.6	63.6	79.0
ETBA model	94.3	77.3	87.7

### Evaluation of the ETBA model with the subgroups validation set

**Figure 5:**
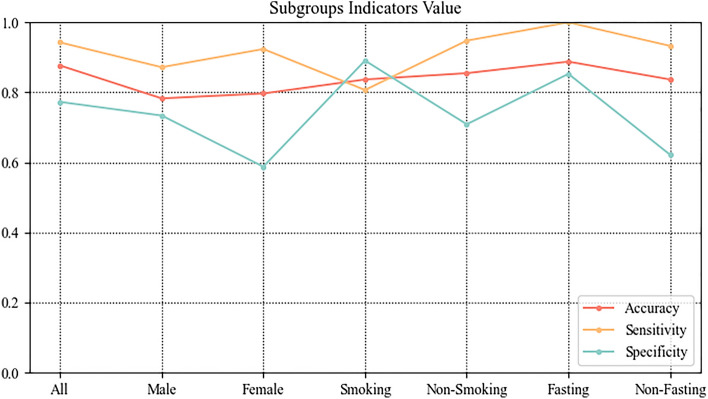
Evaluation of subgroups.

Subgroups were segmented according to demographic characteristics. The male group had a sensitivity of 73.4%, a specificity of 87.2%, and an accuracy of 78.3%. The female group had a sensitivity of 92.4%, a specificity of 58.8%, and an accuracy of 79.7%. The smoking and ex-smoking groups had a sensitivity of 73.4%, a specificity of 87.2%, and an accuracy of 78.3%. The non-smoking group had a sensitivity of 94.8%, a specificity of 70.9%, and an accuracy of 85.5%. The fasting group had a sensitivity of 100%, a specificity of 85.3%, and an accuracy of 88.8%. The non-fasting group had a sensitivity of 93.3%, a specificity of 62.2%, and an accuracy of 83.7%. The evaluation of the subgroups is shown in Fig. 5.

### Feature importance

**Figure 6:**
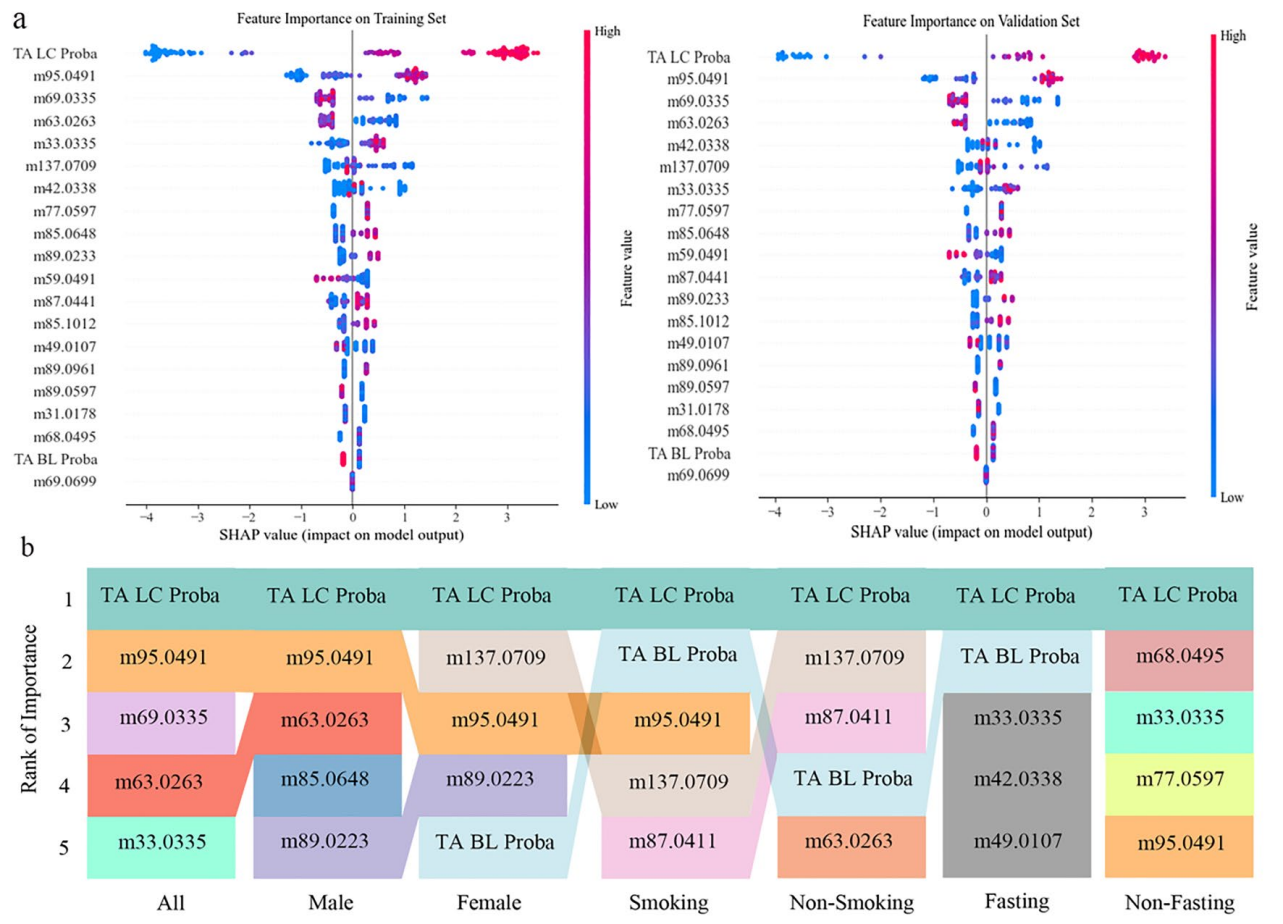
Feature importance. (**a**) Feature importance in the training set and validation set by SHAP. “TA LC Proba” indicates that the LC probability was obtained from the TA model. “TA BL Proba” indicates that the BL probability was obtained from the TA model. (**b**) Feature importance mobility in the subgroup model.

Feature importance refers to the contribution of each VOCs and radiology report to the prediction score. The Shapley additive explanations (SHAP) method was used to evaluate the ETBA model. It indicates the classification capability of each feature. The five most important features for the whole set (training set and validation set) were the LC probability obtained from the TA model, m95.0491, m69.0335, m63.0263, and m33.0335, as shown in Fig. 6a. The feature importance of mobility for the whole set and the different subgroups is shown in Fig. 6b.

## Discussion

BA has the advantage of being non-invasive and rapid. Many studies using BA have investigated different characteristics between LC and healthy controls^18^. PN patients have a greater potential to develop LC than the health participants. Few researchers have developed diagnostic models to identify LC from PNs with good sensitivity^22^. This study used TA on radiology reports combined with BA to construct the ETBA model, which demonstrated good performance.

The results show that BA based on PTR–TOF–MS can be effective in assisting radiologists to provide high-resolution and real-time diagnosis. The TA model, which learned from the report section of the radiology report, demonstrated low-variance and high-sensitivity diagnoses.

Most of the existing TA researchers have explored the classification of tumor types. Inspired by Roxanne Wadia, TA was used to identify LC and BL patients in this study. The TA model’s performance exceeded that of the radiologist diagnosis performance with a sensitivity of 74.3% and a specificity of 77.3%. To construct our highly accurate, multi-dimensional ETBA model, we used the BA model that had a high sensitivity of 88.6% compared with other methods^17^, a specificity of 63.6%, and an accuracy of 79.2%, which was better than the TA model. The ETBA model achieved a sensitivity of 91.4% and a specificity of 86.4%, which inherited and improved the sensitivity of the BA model and the specificity of the TA model. The ETBA model enables multi-modal, high-resolution screening of early-stage LC patients in real-world scenarios, and the result was better than those of other methods that were only based on exhaled breath^21^. In the prediction scores shown in Fig. 4b, 13 BL patients (17 true negative) had scores below 0.3, and 30 LC patients (33 true positive) had scores above 0.7. This indicates that the ETBA model had sufficient discrimination for LC and BL patients. The ETBA model is also capable of diagnosing small, non-solid nodules that are considered to warrant conservative treatment by radiologists.

The leading cause of the improvement in specificity is suspected to be that the ETBA strategy integrates an extra dimension from exhaled breath into the radiology report. BA based on metabolic processes at the tissue level provides features that are not present in the radiology report. BA can identify malignant tumors at the initial stage. For malignant tumors with visible traces, the radiology report provides a clearer description. The ETBA model integrates the advantages of the BA and TA models. As shown in Fig. 4c–h, the tumors that were classified as inflammation by the TA model and radiologist diagnosis were identified correctly by the ETBA model.

To evaluate the ETBA model from multiple perspectives, this study reconstructed models for each subgroup set. For the attribute of gender, the male subgroup’s performance was more stable and concentrated than that of the female subgroup. However, due to sample size limitations, we will collect a larger sample of female subjects in the next stage and find out the reasons for the poor performance of the model in this subgroup. For the attribute of smoking status, the two subgroup models performed consistently and similarly to the model trained with the whole set. For the attribute of dietary status, the fasting subgroup model achieved 100% sensitivity and 85.3% specificity. Another subgroup’s performance was similar to the female subgroup model.

In addition to evaluating the model with statistical analysis, we conducted a feature importance analysis to obtain an in-depth interpretation of the ETBA model and suggestions for future research. According to the feature importance mobility shown in Fig. 6b, m95.0491 appears in the male, female, smoking, and non-fasting subgroups with high weight (second or third rank). In the fasting subgroup, only the probability given by the TA model on the radiology report contributed. However, the BA model also had classification ability in the fasting subgroup, as shown in Extended Data Fig. 1.

The statistical analysis and feature importance analysis both indicate that the ETBA model is effective for assisting LC early-stage screening. Its performance is superior to other existing models that identify LC from PNs and close to that of LC–health control models. In future research, we will perform multi-center calibration to further validate the ETBA model. The limitations of this study will be addressed in the next research stage. We will collect a larger sample of female subjects and conduct deeper analyses to improve the specificity of the female subgroups. The construction of the ETBA model was based on 231 participants. We will use large-scale studies to further calibrate the ETBA model and improve its robustness.

### Supplementary Information

Supplementary Information.

## Data Availability

The data that support the findings of this study are available on request from the corresponding author, sunmx@bme.cams.cn, upon reasonable request.

## References

[1] Siegel RL (2022). Cancer statistics 2022. CA Cancer J. Clin..

[2] Miller KD (2022). Cancer treatment and survivorship statistics, 2022. CA Cancer J. Clin..

[3] Tandberg DJ (2018). Surgery versus stereotactic body radiation therapy for stage I non-small cell lung cancer: A comprehensive review. Cancer.

[4] Shieh Y, Bohnenkamp M (2017). Low-dose CT scan for lung cancer screening: Clinical and coding considerations. Chest.

[5] Petousis P (2016). Prediction of lung cancer incidence on the low-dose computed tomography arm of the National Lung Screening Trial: A dynamic Bayesian network. Artif. Intell. Med..

[6] Li F (2004). Malignant versus benign nodules at CT screening for lung cancer: comparison of thin-section CT findings. Radiology.

[7] Khan A (2022). Lung cancer nodules detection via an adaptive boosting algorithm based on self-normalized multiview convolutional neural network. J. Oncol..

[8] Li Y (2022). Snowflake bionic flow channel design to optimize the pressure drop and flow uniform of proton exchange membrane fuel cells. Micromachines.

[9] Mithun S (2023). Clinical concept-based radiology reports classification pipeline for lung carcinoma. J. Digit. Imag..

[10] Kehl KL (2021). Artificial intelligence-aided clinical annotation of a large multi-cancer genomic dataset. Nat. Commun..

[11] Nobel JM (2020). Natural language processing in dutch free text radiology reports: Challenges in a small language area staging pulmonary oncology. J. Digit. Imag..

[12] Wadia R (2018). Comparison of natural language processing and manual coding for the identification of cross-sectional imaging reports suspicious for lung cancer. JCO Clin. Cancer Inform..

[13] Huang S (2023). Artificial intelligence in lung cancer diagnosis and prognosis: Current application and future perspective. Sem. Cancer Biol..

[14] Mansurova M (2018). A breath of information: The volatilome. Curr. Genet..

[15] Boots AW (2012). The versatile use of exhaled volatile organic compounds in human health and disease. J. Breath Res..

[16] Kort S (2023). Diagnosing non-small cell lung cancer by exhaled breath profiling using an electronic nose: A multicenter validation study. Chest.

[17] Schmidt F (2023). Mapping the landscape of lung cancer breath analysis: A scoping review (ELCABA). Lung Cancer (Amsterdam, Netherlands).

[18] Rudnicka J (2019). Searching for selected VOCs in human breath samples as potential markers of lung cancer. Lung Cancer (Amsterdam, Netherlands)..

[19] Wang P (2022). Identification of lung cancer breath biomarkers based on perioperative breathomics testing: A prospective observational study. EClinicalMedicine.

[20] Temerdashev AZ (2023). Non-invasive lung cancer diagnostics through metabolites in exhaled breath: influence of the disease variability and comorbidities. Metabolites.

[21] Phillips M (2019). A volatile biomarker in breath predicts lung cancer and pulmonary nodules. J. Breath Res..

[22] Chen X (2021). Calculated indices of volatile organic compounds (VOCs) in exhalation for lung cancer screening and early detection. Lung Cancer (Amsterdam, Netherlands).

[23] Peralbo-Molina A (2016). Metabolomics analysis of exhaled breath condensate for discrimination between lung cancer patients and risk factor individuals. J. Breath Res..

[24] Monedeiro F (2021). Needle trap device-GC-MS for characterization of lung diseases based on Breath VOC profiles. Molecules (Basel, Switzerland).

[25] Wang M (2018). Confounding effect of benign pulmonary diseases in selecting volatile organic compounds as markers of lung cancer. J. Breath Res..

