# Supplementary material for: Enhancing diagnosis of benign lesions and lung cancer through ensemble text and breath analysis: a retrospective cohort study
Source: Sci Rep. 2024 Apr 16;14:8731. doi: 10.1038/s41598-024-59474-w (PMC11021445; doi:10.1038/s41598-024-59474-w)
Supplement: Supplementary file 1 — Supplementary Information. [file 41598_2024_59474_MOESM1_ESM.docx]

**Extended Data**

Extended Data Table 1. Twenty-two kinds of VOCs that were selected.

| Mass | Formula | Name | Mass | Formula | Name |
| --- | --- | --- | --- | --- | --- |
| m31.0178 | (CH_2_O)H^+^ | Formaldehyde | m85.1012 | (C_6_H_12_)H^+^ | 1-Hexene |
| m33.0335 | (CH_4_O)H^+^ | Methanol | m87.0441 | (C_4_H_6_O_2_)H^+^ | Biacetyl |
| m42.0338 | (C_2_H_3_N)H^+^ | Acetonitrile | m87.0804 | (C_5_H_10_O)H^+^ | Pentanal |
| m49.0107 | (CH_4_S)H^+^ | Methanethiol | m89.0233 | (C_3_H_4_O_3_)H^+^ | Pyruvic acid |
| m59.0491 | (C_3_H_6_O)H^+^ | Acetone | m89.0419 | (C_4_H_8_S)H^+^ | 1-(Methylsulfanyl)-1-propene |
| m63.0263 | (C_2_H_6_S)H^+^ | (Methylsulfanyl)  methane | m89.0597 | (C_4_H_8_O_2_)H^+^ | 3-Hydroxy-2-butanone |
| m68.0495 | (C_4_H_5_N)H^+^ | Pyrrole | m89.0961 | (C_5_H_12_O)H^+^ | 1-Pentanol |
| m69.0335 | (C_4_H_4_O)H^+^ | Furan | m95.0491 | (C_6_H_6_O)H^+^ | Phenol |
| m69.0699 | (C_5_H_8_)H^+^ | Isoprene | m95.0855 | (C_7_H_10_)H^+^ | Cyclopentylacetylene |
| m77.0597 | (C_3_H_8_O_2_)H^+^ | 1,2-Propanediol | m137.0709 | (C_7_H_8_N_2_O)H^+^ | Anthranilamide |
| m85.0648 | (C_5_H_8_O)H^+^ | Cyclopentanone | m137.1325 | (C_10_H_16_)H^+^ | (4S)-4-Isopropenyl-1-methylcyclohexene |

Extended Data Table 2. Sensitivity, specificity, and accuracy of the models and of the manual diagnosis with the training set.

| Method | Sensitivity | Specificity | Accuracy |
| --- | --- | --- | --- |
| Radiologist diagnosis | 71.4% | 56.5% | 67.8% |
| TA | 89.5% | 89.9% | 90.0% |
| BA | 96.2% | 94.2% | 95.4% |
| ETBA model | 98.1% | 90.0% | 94.8% |


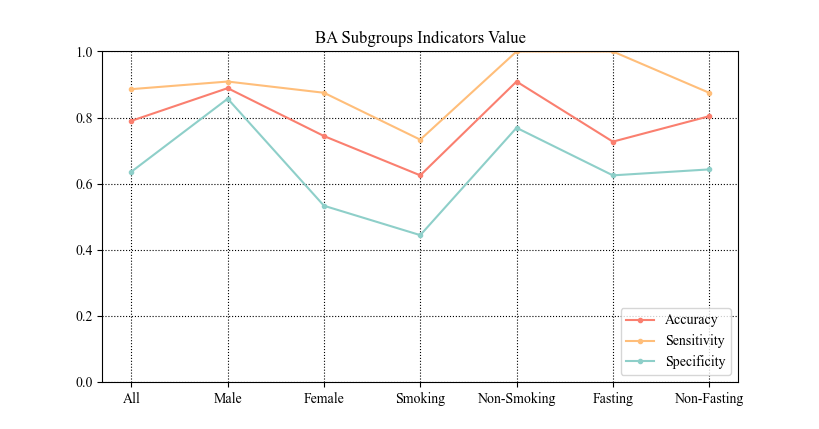


**Extended Data Figure 1. Evaluation of each subgroup with the BA model.**
